# Supplementary material for: Exome sequencing in 16 patients with pituitary stalk interruption syndrome: A monocentric study
Source: PLoS One. 2023 Dec 14;18(12):e0292664. doi: 10.1371/journal.pone.0292664 (PMC10721018; doi:10.1371/journal.pone.0292664)
Supplement: Attachment — Submitted filename: Reviewers.docx [file pone.0292664.s001.docx]

To Reviewer 1

Dear Reviewer,

Thank you for reviewing our manuscript.

As you suggested, the conclusions that “genetic diagnosis of idiopathic PSIS is challenging” of the Abstract and of the Introduction have been completed.

The criteria used to diagnose PSIS are now at the onset of “Patients”. The anterior pituitary gland height, mainly 4 mm, has been indicated. We added to the introduction of the Abstract that “The triad of PSIS can be incomplete”.

The abbreviations of Table 1 have been completed.

To Reviewer 2

Dear Reviewer,

Thank you for reviewing our manuscript.

Concerning the definition of the ACTH deficiency, the values indicated in “Methods” have been completed by the individual values of the 3 patients with ACTH deficiency (below Table 1).

Table 1: Abbreviation section has been completed and the unit of pituitary height added.

As suggested by the other Reviewer, we completed the diagnosis criteria of the PSIS and detailed the pituitary height at the onset of “Patients”. We added to the introduction of the Abstract that “The triad of PSIS can be incomplete”.

Q: What does “MAF and population” mean? I assume that authors noted “minor allele frequency” and its given population. So, the SNP was not detected in other populations?

A: MAF is the minor allelic frequency and it cited population is the population with the highest known frequency of the variant in GnomAD. The variant may well be present in other populations but at lower allelic frequencies.

Q: For the case 1, FANCD2, information of MAF and population were inversed.

A: This has been corrected.

Q: What does “MI” mean?

A: MI is the known mode of inheritance of the disorder associated with the gene. It does not reflect the mode of inheritance in the particular case.

Q: Line 142:” No candidate gene was identified in cases 6,8 and 15.” Does it mean that “no mutation/SNP of the candidate gene”?

A: For clarity this sentence has been modified to “Genetic variants associated with the phenotype could not be identified in candidate genes in the cases 6,8 and 15”
