# Supplementary material for: Exome sequencing in 16 patients with pituitary stalk interruption syndrome: A monocentric study
Source: PLoS One. 2023 Dec 14;18(12):e0292664. doi: 10.1371/journal.pone.0292664 (PMC10721018; doi:10.1371/journal.pone.0292664)
Supplement: Attachment — Submitted filename: To Reviewers.docx [file pone.0292664.s002.docx]

**To Reviewer 1**

Dear Reviewer,

Thank you for reviewing our manuscript. Please find joined the answers to your questions.

4. Have the authors made all data underlying the findings in their manuscript fully available?
Reviewer #1: No

Reviewer #2: Yes

**Answer:** All relevant data are within the manuscript and all novel variants are deposited with ClinVar.

**6. Review Comments to the Author**
Reviewer #1: Given that PLOS ONE’s publication criteria do not include novelty, impact, or interest, the reviewer finds the study's conclusions to be well-founded. However, the reviewer identifies the following issues that need to be addressed.
**Comment 1:**
The diagnostic criteria for PSIS remain unclear. While the authors state that they have provided the diagnostic criteria for PSIS in the opening of the “Patient” section, they presented a clinical description of the patients rather than clear criteria.
A clearer presentation of the diagnostic criteria might look like: “A diagnosis of PSIS was made if the patient met at least ?two? of the following criteria: (1) an absent or ectopic posterior pituitary, (2) an interrupted, malformed, or thin pituitary stalk, and (3) a small anterior pituitary gland.” Based on Table 1 in the original version of the manuscript (which seems to be absent in the revised file), the patient 7 seems to have normal posterior pituitary gland, an interrupted pituitary stalk, and an anterior pituitary with a height of 4 mm. Is her anterior pituitary considered small? Please clarify the reference range for the height of the anterior pituitary gland.

**Answer:** The diagnostic criteria of PSIS have been clarified as you suggested. A pituitary height of 4 mm is normal, as measured in 5 patients and this is indicated in the text as well as the reference with the normal range of the anterior pituitary height.

**Comment** 2:
The reviewer was unable to locate the tables in the revised PDF version of the manuscript.

**Answer:** The two Tables are now included in the text.

**To Reviewer 2**

Dear Reviewer,

Thank you for reviewing our manuscript.

**Comment** 2:
The reviewer was unable to locate the tables in the revised PDF version of the manuscript.

**Answer:** The two Tables are now included in the text.
